# Renin-Angiotensin-Aldosterone System Activation and Diuretic Response in Ambulatory Patients With Heart Failure

**DOI:** 10.1016/j.xkme.2022.100465

**Published:** 2022-04-08

**Authors:** Jonathan G. Amatruda, Rebecca Scherzer, Veena S. Rao, Juan B. Ivey-Miranda, Michael G. Shlipak, Michelle M. Estrella, Jeffrey M. Testani

**Affiliations:** 1 Division of Nephrology, Department of Medicine, University of California, San Francisco, San Francisco, CA; 2 Kidney Health Research Collaborative, San Francisco Veterans Affairs Health Care System & University of California, San Francisco, San Francisco, CA; 3 Department of Internal Medicine, Section of Cardiovascular Medicine, Yale University School of Medicine, New Haven, CT; 4 Hospital de Cardiología, Instituto Mexicano del Seguro Social, Mexico City, Mexico; 5 Department of Medicine, San Francisco Veterans Affairs Health Care System, San Francisco, CA; 6 Division of Nephrology, Department of Medicine, San Francisco VA Health Care System, San Francisco, CA

**Keywords:** Cardiorenal syndrome, diuretic resistance, heart failure, loop diuretics, natriuresis, renin-angiotensin-aldosterone system, torsemide

## Abstract

**Rationale & Objective:**

Heart failure treatment relies on loop diuretics to induce natriuresis and decongestion, but the therapy is often limited by diuretic resistance. We explored the association of renin-angiotensin-aldosterone system (RAAS) activation with diuretic response.

**Study Design:**

Observational cohort.

**Setting & Population:**

Euvolemic ambulatory adults with chronic heart failure were administered torsemide in a monitored environment.

**Predictors:**

Plasma total renin, active renin, angiotensinogen, and aldosterone levels. Urine total renin and angiotensinogen levels.

**Outcomes:**

Sodium output per doubling of diuretic dose and fractional excretion of sodium per doubling of diuretic dose.

**Analytical Approach:**

Robust linear regression models estimated the associations of each RAAS intermediate with outcomes.

**Results:**

The analysis included 56 participants, and the median age was 65 years; 50% were women, and 41% were Black. The median home diuretic dose was 80-mg furosemide equivalents. In unadjusted and multivariable-adjusted models, higher levels of RAAS measures were generally associated with lower diuretic efficiency. Higher plasma total renin remained significantly associated with lower sodium output per doubling of diuretic dose (β = −0.41 [−0.76, −0.059] per SD change) with adjustment; higher plasma total and active renin were significantly associated with lower fractional excretion of sodium per doubling of diuretic dose (β = −0.48 [−0.83, −0.14] and β = −0.51 [−0.95, −0.08], respectively) in adjusted models. Stratification by RAAS inhibitor use did not substantially alter these associations.

**Limitations:**

Small sample size; highly selected participants; associations may not be causal.

**Conclusions:**

Among multiple measures of RAAS activation, higher plasma total and active renin levels were consistently associated with lower diuretic response. These findings highlight the potential drivers of diuretic resistance and underscore the need for high-quality trials of decongestive therapy enhanced by RAAS blockade.

## Visual Abstract

**Figure:**
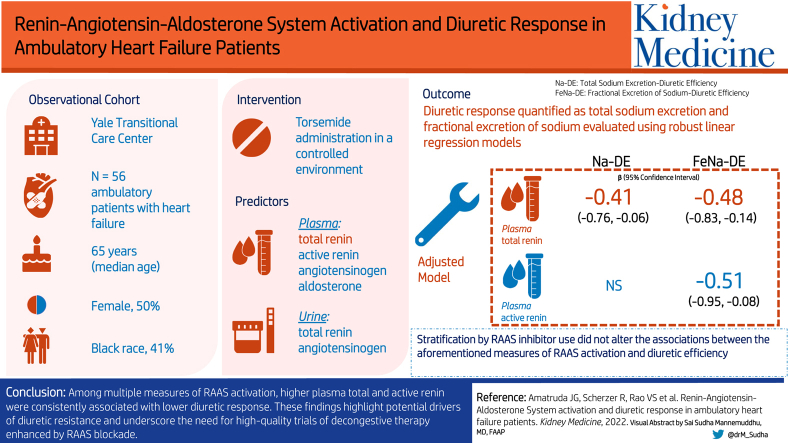

**Plain-Language Summary**

Loop diuretics constitute the foundation of heart failure treatment, but effective therapy is often hindered by diuretic resistance. Renin-angiotensin-aldosterone system (RAAS) activation may play a role in diuretic response by driving sodium and fluid retention despite loop diuretic use. We investigated the association of RAAS activation with diuretic response among ambulatory adults with heart failure undergoing monitored diuresis. We measured RAAS intermediates in plasma and urine and then estimated their associations with measures of sodium excretion standardized to a diuretic dose. In multivariable-adjusted models, plasma total renin and active renin appeared to be consistently associated with diuretic response. The findings suggest that RAAS activation may play a role in diuretic resistance and may help clinicians tailor therapy for patients with heart failure.

Effective diuresis in heart failure is associated with fewer hospitalizations and lower mortality; however, inadequate loop diuretic response poses a clinical challenge.^1^, ^2^, ^3^, ^4^ This problem has generally been described as “diuretic resistance,” referring to limited diuresis or natriuresis despite presumably adequate diuretic dose.^4^ Unfortunately, diuretic resistance is common and portends worse outcomes.^5^^,^^6^ Therefore, a better understanding of the mechanisms underlying differences in diuretic response is integral to improving care for patients with heart failure.

The variation in the diuretic response is driven in part by pathways modulating volume and salt homeostasis. The renin-angiotensin-aldosterone system (RAAS) is a key component of the neurohormonal axis responsible for maintaining blood pressure by modulating intravascular volume, salt retention, and vascular tone—all central mechanisms of disease in heart failure.^4^^,^^7^ Drugs targeting the RAAS intermediates, such as angiotensin-converting enzyme inhibitors, angiotensin receptor blockers (ARBs), and mineralocorticoid receptor antagonists (MRAs), can improve heart failure symptoms and outcomes and are widely employed in this population.^8^ However, less is known about the extent to which RAAS activation relates to the treatment of heart failure, particularly with loop diuretics. Few studies have investigated whether pharmacologic RAAS inhibition improves loop diuretic response, and these have yielded mixed results.^9^, ^10^, ^11^, ^12^

To further explore this relationship, we analyzed associations between indicators of RAAS activation and detailed measures of diuretic response in an ambulatory cohort of patients with heart failure undergoing monitored diuretic therapy at the Yale Transitional Care Center (YTCC). In these participants, we measured renin, angiotensinogen, and aldosterone levels. The action of renin on angiotensinogen can be considered a rate-determining step in the RAAS, and their levels can indicate RAAS activation.^13^, ^14^, ^15^ Aldosterone—the final product of the RAAS—drives sodium reabsorption and is a key mediator in the pathophysiology of heart failure.^8^^,^^16^^,^^17^ We hypothesized that higher prediuretic levels of renin, angiotensinogen, and aldosterone would be associated with lower diuretic response.

## Methods

### Study Design and Cohort

The analytic cohort consisted of prospectively enrolled ambulatory patients with heart failure who were treated at the YTCC and has been previously described.^18^ The study was approved by the institutional review board at the Yale School of Medicine, and all participants provided written informed consent (HIC# 1212011229). The study design complied with the Declaration of Helsinki.

Participants presented to the YTCC for posthospitalization follow-up or as referrals for volume management; those deemed clinically euvolemic were given oral torsemide at doses equivalent to their home diuretics to assess for diuretic response. All participants were instructed not to take their scheduled morning diuretic before presenting at the YTCC. The study staff recorded prediuretic vital signs and obtained blood and urine samples. After the diuretic administration, the urine output was recorded, and additional blood and urine samples were collected approximately 1.5 hours after diuretic administration, corresponding to the time of peak diuresis. The 56 participants who received torsemide at the YTCC and had no missing observations for RAAS measures or outcomes comprised the analytical cohort for this study.

### Laboratory Measurements and Assays

Urine and serum electrolytes were measured on a Randox RxDaytona automated clinical chemistry analyzer using ion-selective electrodes (Randox Laboratories Ltd). Commercially available urine and serum controls from Randox Laboratories were run concurrently with each batch of samples. Creatinine was measured using Randox reagents as per the manufacturer’s instructions. Creatinine measurements were standardized to the National Institute of Standards and Technology reference material (SRM 967).

### Exposures

Biomarkers of RAAS activation were measured in both plasma and urine. In plasma, we measured total renin, active renin, angiotensinogen, and aldosterone levels; these samples were obtained from participants who had been in the seated position for approximately 1.5-2 hours. In urine, we measured total renin and angiotensinogen levels, which were indexed to urine creatinine to account for variations in urine concentration. Urine measurements were chosen in addition to plasma because the presence of these intermediates in urine is hypothesized to reflect intrarenal RAAS activation, which may be particularly relevant to diuretic response.^13^ Total renin and angiotensinogen levels were measured using 2-plex prototype kits from Meso Scale Discovery (MSD, Meso Scale Diagnostics, LLC). The electrochemiluminescence from the plate was read using the MSD Quickplex SQ120 reader (Meso Scale Discovery). The mean lower limit of detection was 1.25 pg/mL for renin and 15.10 pg/mL for angiotensinogen. The assay for total renin recognizes both prorenin and active renin. The plasma aldosterone level was measured by radioimmunoassay kit (Alpco) as per manufacturer’s instructions.

### Outcomes

The diuretic response was evaluated using 2 measures of diuretic efficiency (DE) based on natriuresis, which have been used in prior studies of diuretic response: total sodium excreted per 2-fold higher diuretic dose (Na-DE) and fractional excretion of sodium at peak diuresis per 2-fold higher diuretic dose (FENa-DE).^19^ Total sodium excretion represented the total amount of sodium in the urine in millimoles over the course of the postdiuretic urine collection period and was calculated by multiplying the urine sodium concentration in a spot urine sample by the total volume of urine produced during urine collection. The fractional excretion of sodium was measured from samples obtained at peak diuresis and was calculated as follows:

*Fractional excretion of sodium = [urine (sodium) × plasma (creatinine)]/[plasma (sodium) × urine (creatinine)] × 100%*

DE represents a measure of natriuresis standardized to diuretic dose, as natriuresis is expected to vary with the loop diuretic dose even in diuretic resistant individuals.^20^ Na-DE and FENa-DE are both measures of sodium excretion accounting for diuretic dose. However, FENa-DE accounts for variations in nephron mass because fractional excretion of sodium is influenced by glomerular filtration rate to a lesser extent than total urine sodium.^19^ To account for the log-linear dose-response curve of loop diuretics, the diuretic dose was log_2_-transformed to represent each 2-fold higher diuretic dose in furosemide equivalents.^18^^,^^19^ All DE calculations were centered on a dose of 40-mg intravenous furosemide equivalents. Doses ≤ 20-mg furosemide equivalents were winsorized to zero to prevent the generation of negative numbers. The DE calculations used were as follows:

*Na-DE = [total Na output]/[log*_*2*_ *(furosemide equivalents administered) − 4.32]*

*FENa-DE = [fractional excretion of sodium at peak diuresis]/[log*_*2*_ *(furosemide equivalents administered) −4.32]*

To determine equipotent loop diuretic doses, we used the following conversion: 1-mg bumetanide = 20-mg torsemide = 40-mg intravenous furosemide = 80-mg oral furosemide.

### Covariates

Demographic covariates included age, sex, and race. Clinical covariates included prevalent diabetes mellitus, systolic blood pressure at presentation to YTCC, serum albumin level, estimated glomerular filtration rate (eGFR) based on the CKD-EPI (Chronic Kidney Disease Epidemiology Collaboration) equation, and serum N-terminal pro–B-type natriuretic peptide (NT-proBNP) level.^21^ All measured covariates were from prediuretic urine samples and observations. In addition, medications affecting the RAAS axis, such as the angiotensin-converting enzyme inhibitors, ARBs, and MRAs, were included as covariates.

### Statistical Analysis

Descriptive statistics were tabulated, and values were reported as medians (interquartile ranges) or numbers (%). All biomarkers and outcomes were log-transformed to normalize skewed distributions. We used Spearman ρ to evaluate correlations between continuous RAAS biomarkers and key covariates. Model checking included testing for the normality of the error residuals and testing for heteroscedasticity. Because influential outliers were identified in some models, we used robust linear regression models using modified maximum (MM) likelihood type estimation to estimate the association of each neurohormone with each measure of natriuresis.^22^ We adjusted for covariates in 2 steps: model 1 adjusted for demographics; model 2 further adjusted for prevalent diabetes, serum albumin level, eGFR, NT-proBNP level, and use of angiotensin-converting enzyme inhibitors, ARBs, and MRAs. Coefficients were standardized to represent per standard deviation effect. All analyses were conducted using the SAS system, version 9.4 (SAS Institute, Inc).

## Results

**Figure 1:**
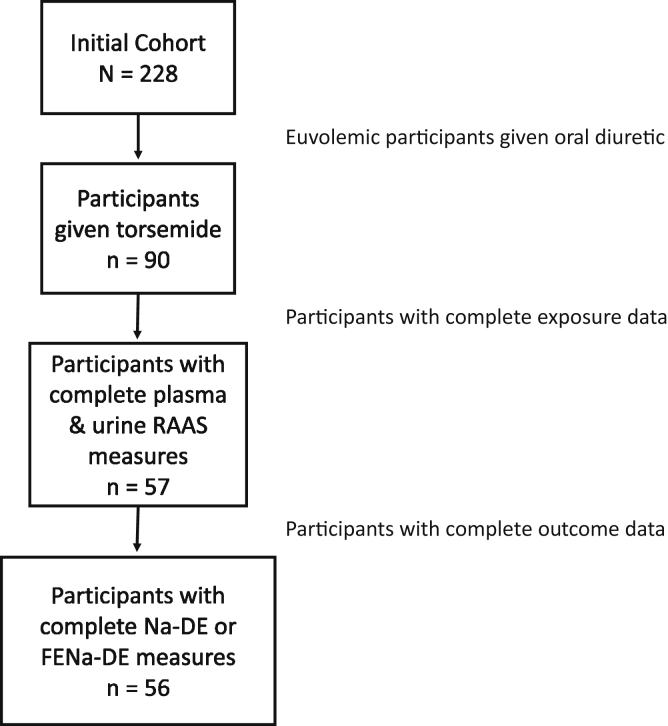
Flow chart depicting the sampling of initial YTCC cohort to produce final analytic sample. Abbreviations: FENa-DE, fractional excretion of sodium at peak diuresis per 2-fold higher diuretic dose; Na-DE; total sodium excreted per 2-fold higher diuretic dose; RAAS, renin-angiotensin-aldosterone system; YTCC, Yale Transitional Care Center.

Of the 228 participants enrolled at the YTCC, 90 were given torsemide. Of the participants given torsemide, 57 had RAAS intermediates measured, 56 of whom had Na-DE or fractional excretion of sodium measured (Fig 1). At baseline, the final analytic sample of 56 participants had a median age of 65 years, 50% were women, and 41% were Black (Table 1). The median left ventricular ejection fraction was 37%, with median serum NT-proBNP level of 1,385 pg/mL and median eGFR of 52 mL/min/1.73 m^2^. The median home diuretic dose was 80-mg furosemide equivalents. At the time of study enrollment, 55% of participants used angiotensin-converting enzyme inhibitors or ARBs; 32% used MRAs.

**Figure 2:**
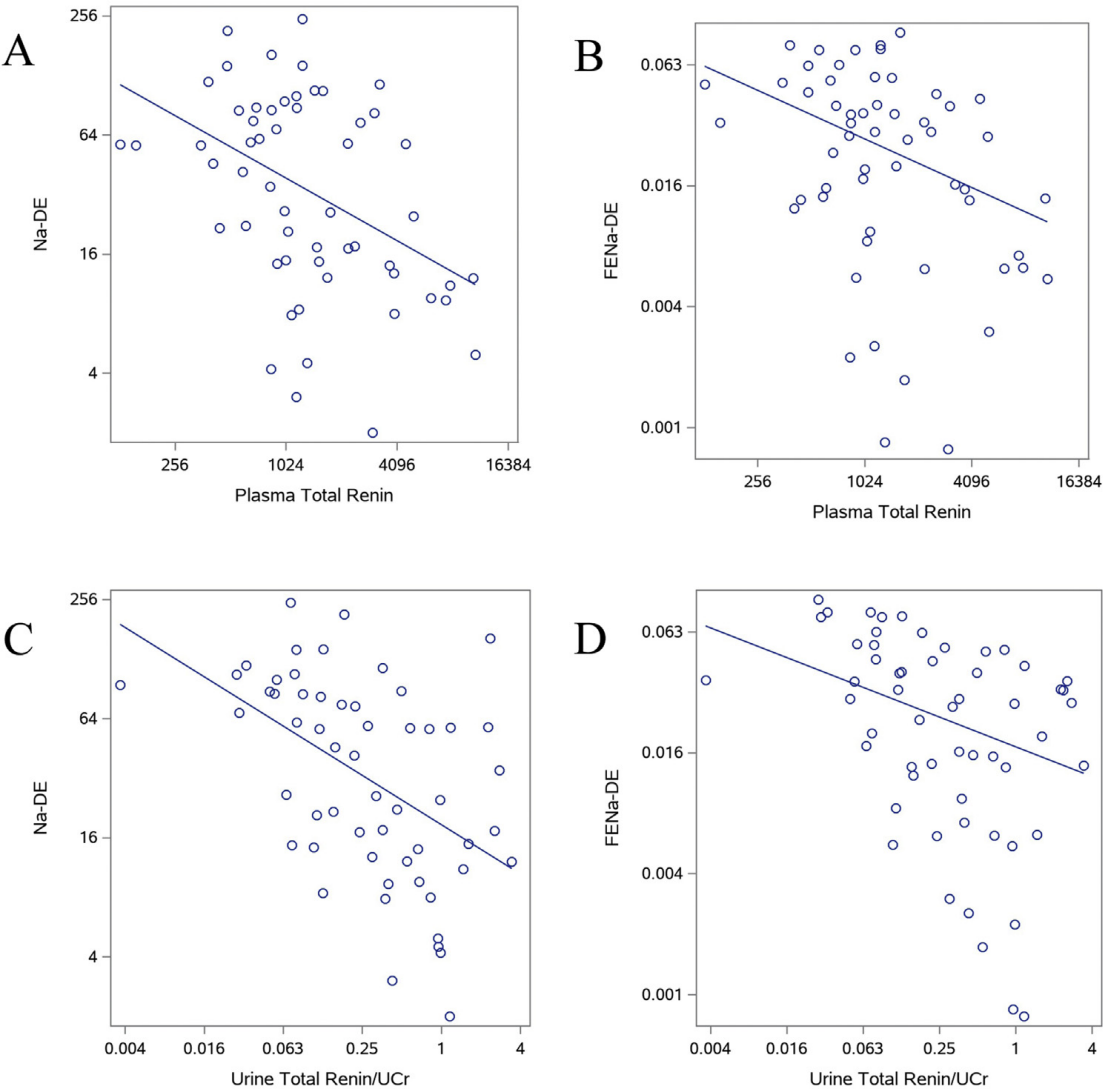
Scatterplots of associations of plasma and urine total renin with Na-DE and FENa-DE. Scatterplots and lines of best fit for associations of (A) plasma total renin with Na-DE, (B) plasma total renin with FENa-DE, (C) urine total renin indexed to urine creatinine with Na-DE, and (D) urine total renin indexed to urine creatinine with FENa-DE. All outcomes and biomarkers are log_2_-transformed. Fitted lines from robust regression models use modified maximum likelihood estimation. Abbreviations: FENa-DE, fractional excretion of sodium at peak diuresis per 2-fold higher diuretic dose; Na-DE; total sodium excreted per 2-fold higher diuretic dose.

**Table 1:** Demographic and Baseline Clinical Characteristics Overall and Stratified by Median Fractional Excretion of Sodium Per Doubling of Diuretic Dose

Characteristic	All (N = 56)	FENa-DE < Median (n = 28)	FENa-DE ≥ Median (n = 28)
Age, y	65 (57-74)	65 (58-74)	65 (57-73)
Female sex	28 (50%)	12 (43%)	15 (54%)
Black race	23 (41%)	6 (21%)	16 (57%)
Home diuretic dose, furosemide equivalents	80 (40-160)	120 (40-160)	80 (40-120)
Administered diuretic dose, furosemide equivalents	40 (20-120)	80 (35-160)	40 (20-60)
Systolic blood pressure, mm Hg	126 (108-138)	3.9 (3.6-4.1)	4.1 (3.7-4.2)
Diabetes mellitus	28 (50%)	128 (109-136)	124 (110-142)
Left ventricular ejection fraction, %	37 (23-55)	17 (61%)	11 (39%)
eGFR, mL/min/1.73 m^2^	52 (32-79)	40 (21-58)	42 (26-53)
Serum albumin level, g/dL	3.9 (3.7-4.1)	47 (27-73)	68 (37-87)
NT-proBNP level, pg/mL	1,385 (467-3,540)	1,260 (675-4,500)	1,280 (364-3,315)
Urine albumin-to-creatinine ratio, mg/g	21 (10-103)	17 (14-96)	25 (9-81)
ACEi or ARB use	31 (55%)	14 (50%)	17 (61%)
Mineralocorticoid receptor antagonist use	18 (32%)	9 (32%)	8 (29%)

*Note*: Data are median (interquartile range) or number (percentage). Median FENa-DE = 0.0278.

Abbreviations: ACEi, angiotensin-converting enzyme inhibitor; ARB, angiotensin receptor blocker; eGFR, estimated glomerular filtration rate; FENa-DE, fractional excretion of sodium at peak diuresis per 2-fold higher diuretic dose; NT-proBNP, N-terminal pro–brain natriuretic peptide.

Spearman correlations of the urine and plasma RAAS measures with each other and with eGFR are presented in Table S1. Plasma total renin, active renin, and angiotensinogen were moderately correlated with each other and each was modestly correlated with aldosterone (Table S1). In general, aldosterone was more weakly correlated with the other urine and plasma RAAS measures. Higher eGFR correlated with lower levels of all RAAS measures, and these correlations were statistically significant for the urine biomarkers (urine angiotensinogen, *r* = −0.43, *P* = 0.001; urine total renin, *r* = −0.32, *P* = 0.02; urine ACR, *r* = −0.32, *P* = 0.02) and plasma total renin (*r* = −0.27, *P* = 0.04).

### Association of RAAS Measures With Na-DE

In the unadjusted model, higher levels of all urine and plasma RAAS measures appeared to be associated with lower Na-DE, although the associations of urine and plasma angiotensinogen with Na-DE were not statistically significant (Table 2). Urine total renin, plasma total renin, plasma active renin, and plasma aldosterone demonstrated comparable effect sizes in their unadjusted associations with Na-DE. In the fully adjusted model, only plasma total renin maintained a significant association with Na-DE, and the point estimate was only modestly attenuated by adjustment (Table 2; Fig 2).

**Table 2:** Associations of Urine and Plasma RAAS Measures With Diuretic Efficiency After Torsemide Administration

RAAS Measure	Model 1	Model 2
Per SD change	β (95% CI)	β (95% CI)
	Total Sodium Excretion-Diuretic Efficiency	
Urine total renin	**−**0.53 (−0.81 to −0.24)	**−**0.25 (**−**0.55 to 0.044)
Plasma total renin	−0.48 (−0.77 to −0.19)	−0.41 (−0.76 to −0.059)
Plasma active renin	−0.55 (−0.85 to −0.24)	**−**0.36 (**−**0.79 to 0.074)
Urine angiotensinogen	**−**0.22 (**−**0.53 to 0.091)	0.14 (**−**0.20 to 0.49)
Plasma angiotensinogen	**−**0.19 (**−**0.52 to 0.15)	0.00 (**−**0.34 to 0.34)
Plasma aldosterone	−0.49 (−0.75 to −0.22)	**−**0.29 (**−**0.73 to 0.16)

	Fractional Excretion of Sodium-Diuretic Efficiency	
Urine total renin	−0.32 (−0.59 to −0.055)	**−**0.31 (**−**0.64 to 0.023)
Plasma total renin	−0.38 (−0.63 to −0.13)	−0.48 (−0.83 to −0.14)
Plasma active renin	−0.40 (−0.69 to −0.12)	−0.51 (−0.95 to −0.080)
Urine angiotensinogen	**−**0.068 (**−**0.35 to 0.21)	**−**0.051 (**−**0.42 to 0.32)
Plasma angiotensinogen	**−**0.12 (**−**0.41 to 0.16)	**−**0.017 (**−**0.40 to 0.36)
Plasma aldosterone	−0.31 (−0.56 to −0.060)	**−**0.25 (**−**0.74 to 0.24)

*Note*: Urine measures indexed to urine creatinine. Model 1: unadjusted. Model 2: adjusted for age, sex, race, baseline diabetes, serum albumin, estimated glomerular filtration rate (prediuretic), systolic blood pressure, log (N-terminal pro–brain natriuretic peptide), angiotensin-converting enzyme inhibitor use, angiotensin receptor blocker use, and mineralocorticoid receptor antagonist use.

Abbreviations: CI, confidence interval; RAAS, renin-angiotensin-aldosterone system; SD, standard deviation.

In sensitivity analyses stratified on the use of (1) any RAAS inhibitor, (2) angiotensin-converting enzyme inhibitors or ARBs, and (3) MRAs, results were substantively similar (Tables S2-S4). The effect sizes were similar across strata, and there was no strong evidence for interactions on any of the 3 strata with Na-DE.

### Association of RAAS Measures With FENa-DE

Higher levels of all urine and plasma RAAS measures were associated with lower FENa-DE in unadjusted and fully adjusted models (Table 2; Fig 1). While urine total renin, plasma total renin, plasma active renin, and aldosterone were significantly associated with lower FENa-DE at peak diuresis in the unadjusted models, only plasma total and active renin remained significantly associated with FENa-DE in the adjusted models. Notably, the effect sizes for both plasma total and active renin were modestly larger after adjustment.

When stratified by the use of medications that inhibit the RAAS, there was no statistically significant evidence for interactions (Tables S2-S4).

## Discussion

Using multiple measures of RAAS activation and diuretic response, we found that higher levels of plasma total renin were associated with reduced natriuresis among clinically euvolemic patients with heart failure using 2 diuretic response measures, independent of demographic and clinical characteristics. Plasma active renin, urine total renin, and aldosterone also appeared to be associated with lower DE, although these associations did not consistently reach statistical significance in adjusted models. However, there was little evidence for associations of plasma or urine angiotensinogen levels with DE. Likewise, we did not find compelling evidence that associations of the RAAS measures with DE are substantially modified by the use of drugs that inhibit the RAAS. These findings raise the possibility that RAAS activation could explain some of the variability in the diuretic response among patients with heart failure.

Among patients with heart failure, poor diuretic response complicates treatment and affects long-term outcomes. A better understanding of diuretic response and the drivers of diuretic resistance may facilitate strategies to improve decongestive therapy. Much of the existing literature has focused on the role of RAAS activation in the pathophysiology of heart failure, but comparatively less emphasis has been placed on the question of how the RAAS affects the treatment of heart failure. RAAS-mediated antinatriuresis may blunt the diuretic response and contribute to the “braking phenomenon” that countervails prolonged diuresis.^7^ Angiotensin II and aldosterone increase the sodium uptake in the proximal and distal tubules. This compensatory sodium reabsorption, especially in the distal nephron, appears to be a major determinant of loop diuretic response.^19^^,^^23^ Intense loop diuretic exposure stimulates aldosterone production, which augments distal nephron sodium reabsorption through multiple mechanisms, including upregulation of epithelial sodium channel and pendrin activity as well as potentially increasing the sodium-chloride cotransporter abundance.^24^, ^25^, ^26^, ^27^

Although the RAAS has been physiologically implicated in diuretic response, the clinical relevance of this relationship remains uncertain because previous studies have had key methodological limitations. In patients with heart failure, higher levels of plasma aldosterone appear to correlate with loop diuretic resistance, which may be mitigated by angiotensin-converting enzyme inhibitors and spironolactone administration, although these data derive primarily from small observational studies.^10^^,^^28^ The Aldosterone Targeted Neurohormonal Combined with Natriuresis Therapy in Heart Failure (ATHENA-HF) trial randomized adults with heart failure undergoing diuresis to spironolactone 100 mg daily or usual care and found no benefit attributable to spironolactone even among those with lower eGFR or risk factors for diuretic resistance.^11^^,^^29^ However, the ATHENA-HF trial did not assess loop diuretic resistance and has been criticized for its short duration of treatment and absence of metabolic evidence of effective spironolactone dosing.^30^ In a more recent trial, high-dose spironolactone appeared to improve diuretic efficacy among patients with acute heart failure exhibiting resistance to intravenous furosemide, but this intervention was not randomized or placebo-controlled and was again limited by short duration of spironolactone treatment.^12^

Plasma total renin, which represents both active renin and prorenin, was most consistently associated with diuretic response in our analysis. Higher prediuretic levels of plasma total renin were associated with less natriuresis, and these associations remained robust after adjustment for clinical and pharmacologic covariates and were substantively unchanged after stratification by RAAS inhibitor use. The associations between plasma active renin and natriuresis were comparable to those of total renin but were less precise. Higher urine total renin was also associated with less natriuresis but did not remain statistically significant with adjustment. The associations of higher renin with lower sodium excretion supported our hypothesis and agreed with the existing evidence of poorer outcomes for heart failure patients with higher renin levels.^31^ RAAS activation, as indicated by plasma renin levels, may be one mechanism through which diuretic resistance associates with worse prognosis. Notably, renin release is the initial and rate-limiting step in RAAS activation, which may explain its consistent associations with natriuresis in this analysis. Despite renin’s foundational role in RAAS activation, whether direct renin inhibition offers clinical benefit over other means of interrupting the renin-angiotensin-aldosterone axis is unclear. Aliskiren, the only currently available direct renin inhibitor, is approved for the treatment of hypertension but has not been widely adopted outside niche contexts. Studies of aliskiren in murine heart failure models and healthy humans suggest that it may enhance natriuresis and improve volume status.^32^^,^^33^ However, randomized clinical trials of aliskiren in patients with heart failure, despite showing improvements in biomarkers associated with heart failure, have failed to demonstrate improvements in outcomes such as cardiovascular death or hospitalization.^34^, ^35^, ^36^, ^37^

We found little evidence for associations of plasma or urine angiotensinogen levels with diuretic response. Angiotensinogen, like renin, participates in the most upstream step of the RAAS and is understood to be an indicator of overall RAAS activity.^14^ The plasma angiotensinogen level reflects the systemic RAAS activity, which shapes human heart failure through multiple pathways.^15^ The urine angiotensinogen level is recognized as a marker of local intrarenal RAAS activation, which may be particularly important in heart failure and diuretic response given its proximity to the machinery of salt reabsorption. Compared with angiotensin II concentration in the circulation, angiotensin II concentrations within the kidney are extremely high, and angiotensin II in the renal tissue appears to be locally produced and active.^16^ Animal models of heart failure demonstrate renal angiotensinogen mRNA expression consistent with RAAS activation in heart failure;^38^, ^39^, ^40^ the urine angiotensinogen level may likewise represent intrarenal RAAS activation, which could make it a useful biomarker in heart failure.^41^^,^^42^ However, there remains limited evidence for the clinical relevance of this pathway, and our analyses did not provide additional support for these hypotheses.

Aldosterone is widely accepted as a key factor in the pathophysiology and natural history of heart failure, directly stimulating sodium reabsorption in the kidney and instigating the inflammation and fibrosis leading to myocardial remodeling and heart failure progression.^43^ Prevention of aldosterone’s deleterious effects could explain the success of angiotensin-converting enzyme inhibitors, ARBs, and MRAs in improving heart failure outcomes.^8^ However, RAAS inhibitors may incompletely block the effects of RAAS mediators and paradoxically increase the production of renin, fueling a phenotype of “aldosterone breakthrough.”^44^^,^^45^ In this analysis, we found that higher aldosterone levels may be associated with less sodium excretion, but estimates did not reach statistical significance in the adjusted models. Furthermore, RAAS activation is suspected to have a more prominent role in diuretic resistant patients; expanding this question to a larger population may better elucidate the role of the RAAS in diuretic response.^46^

Our study had several important limitations. The small sample size may have limited the ability to detect meaningful associations and to directly compare RAAS measures to one another. Because renin levels increase with volume depletion, the observed associations between plasma renin concentrations and DE may not be causal.^47^ Moreover, the observational design of this study precluded the ability to make causal claims based on these data. For example, participants with lower renin levels may have greater volume overload and thus be more likely to have a robust diuretic response, whereas more volume-depleted participants may have had appropriate RAAS activation and would also be less likely to respond to diuretics. Although all participants in this analysis were deemed euvolemic by an examining cardiologist, the clinical examination may have been insufficiently sensitive to meaningful differences in the volume status. Although this study modeled associations of several exposures with 2 outcomes, we did not formally adjust for multiple comparisons because our analysis had a single global hypothesis that measures of RAAS activation would be associated with a reduced response to diuretics. We hypothesized *a priori* that all biomarkers would show directionally consistent associations with the outcome, demonstrating a mutually reinforcing pattern rather than a series of independent tests. Another limitation was that the YTCC cohort is a selected group of closely monitored patients with heart failure who may not represent average patients with heart failure in the community. Although we took care to adjust for measured confounders, this observational study was susceptible to residual confounding. Finally, our results do not exclude a role for intrarenal RAAS activation as an important driver of diuretic response in heart failure. The most optimal methods to measure urine renin and urine angiotensinogen are uncertain. The measurement of urine markers is challenging in the setting of diuretic therapy and may require a larger sample to demonstrate underlying patterns.

In conclusion, we found that multiple measures of RAAS activation demonstrated consistent associations with DE after torsemide administration among outpatients with heart failure. Importantly, the associations of these neurohormones with sodium excretion were apparent irrespective of the use of medications that interfere with the RAAS pathway, suggesting that other interventions may be necessary to improve the diuretic response if RAAS activation is driving sodium retention. Future research should aim to reproduce these findings in a larger group of heart failure patients and to evaluate candidate methods to mitigate RAAS activation to improve diuretic response and long-term prognosis.
