# Supplementary material for: Renin-Angiotensin-Aldosterone System Activation and Diuretic Response in Ambulatory Patients With Heart Failure
Source: Kidney Med. 2022 Apr 8;4(6):100465. doi: 10.1016/j.xkme.2022.100465 (PMC9127684; doi:10.1016/j.xkme.2022.100465)
Supplement: Supplementary File 1 (PDF) — Tables S1-S4. [file mmc1.pdf]

**Table S1. Unadjusted Correlations of Urine and Plasma RAAS Intermediates and eGFR.**

| | Urine angiotensinogen | Plasma angiotensinogen | Urine total renin | Plasma total renin | Plasma active renin | Aldosterone | eGFR |
|---|---|---|---|---|---|---|---|
| Urine angiotensinogen | 1 | | | | | | |
| Plasma angiotensinogen | 0.0091 | 1 | | | | | |
| Urine total renin | 0.50** | 0.012 | 1 | | | | |
| Plasma total renin | -0.0078 | 0.46** | 0.35** | 1 | | | |
| Plasma active renin | 0.054 | 0.62** | 0.22 | 0.68** | 1 | | |
| Aldosterone | 0.068 | 0.27* | 0.19 | 0.26 | 0.33* | 1 | |
| eGFR | -0.43** | -0.12 | -0.32* | -0.27* | -0.17 | -0.18 | 1 |

Urine measures indexed to urine creatinine.
eGFR: estimated glomerular filtration rate.
*p <0.05
**p <0.01

**Table S2. Associations of Urine and Plasma RAAS Measures with Sodium-Diuretic Efficiency and Fractional Excretion of Sodium-Diuretic Efficiency after Torsemide Administration in 56 Participants with Heart Failure stratified by RAAS inhibitor* use**

| | Total Sodium Excretion-Diuretic Efficiency | | | | | |
|---|---|---|---|---|---|---|
| | RAASI non-users | | RAASI users | | User vs. non-user interaction P-value | |
| RAAS measure (per SD change) | Model 1 ß (95% CI) | Model 2 ß (95% CI) | Model 1 ß (95% CI) | Model 2 ß (95% CI) | Model 1 P-value | Model 2 P-value |
| Urine total renin | **-0.49 (-0.90, -0.080)** | -0.31 (-0.78, 0.16) | **-0.55 (-0.95, -0.15)** | -0.27 (-0.68, 0.13) | 0.84 | 0.90 |
| Plasma total renin | **-0.44 (-0.87, -0.0085)** | **-0.50 (-0.95, -0.037)** | **-0.61 (-1.01, -0.21)** | -0.28 (-0.79, 0.24) | 0.57 | 0.51 |
| Plasma active renin | -0.59 (-1.19, 0.012) | -0.47 (-1.12, 0.18) | **-0.50 (-0.87, -0.13)** | -0.32 (-0.84, 0.20) | 0.81 | 0.69 |
| Urine angiotensinogen | -0.13 (-0.58, 0.33) | 0.0070 (-0.46, 0.47) | -0.26 (-0.72, 0.21) | 0.14 (-0.35, 0.63) | 0.70 | 0.67 |
| Plasma angiotensinogen | -0.12 (-0.70, 0.46) | -0.16 (-0.74, 0.42) | -0.26 (-0.69, 0.17) | -0.021 (-0.43, 0.38) | 0.70 | 0.68 |
| Plasma aldosterone | -0.64 (-1.36, 0.085) | -0.37 (-1.15, 0.41) | **-0.46 (-0.75, -0.18)** | -0.32 (-0.67, 0.019) | 0.66 | 0.91 |

| | Fractional Excretion of Sodium-Diuretic Efficiency | | | | | |
|---|---|---|---|---|---|---|
| | RAASI non-users | | RAASI users | | User vs. non-user interaction P-value | |
| RAAS measure (per SD change) | Model 1 ß (95% CI) | Model 2 ß (95% CI) | Model 1 ß (95% CI) | Model 2 ß (95% CI) | Model 1 P-value | Model 2 P-value |
| Urine total renin | -0.22 (-0.62, 0.18) | -0.33 (-0.82, 0.17) | **-0.41 (-0.78, -0.039)** | -0.33 (-0.76, 0.097) | 0.49 | 0.99 |
| Plasma total renin | -0.37 (-0.76, 0.027) | **-0.55 (-1.02, -0.086)** | **-0.44 (-0.80, -0.081)** | -0.39 (-0.87, 0.087) | 0.78 | 0.63 |
| Plasma active renin | **-0.69 (-1.26, -0.12)** | **-0.80 (-1.45, -0.15)** | -0.32 (-0.65, 0.0020) | -0.42 (-0.90, 0.056) | 0.28 | 0.32 |
| Urine angiotensinogen | -0.029 (-0.44, 0.38) | -0.072 (-0.56, 0.42) | -0.10 (-0.53, 0.33) | -0.074 (-0.59, 0.45) | 0.81 | 0.99 |
| Plasma angiotensinogen | -0.21 (-0.73, 0.30) | -0.46 (-1.05, 0.14) | -0.083 (-0.46, 0.29) | 0.094 (-0.32, 0.51) | 0.69 | 0.12 |
| Plasma aldosterone | **-0.78 (-1.49, -0.064)** | -0.56 (-1.40, 0.27) | -0.23 (-0.51, 0.058) | -0.19 (-0.55, 0.17) | 0.16 | 0.42 |

*RAAS inhibitors include ACE inhibitors, ARBs, MRAs.
Urine measures indexed to urine creatinine. SD: standard deviation. CI: confidence interval.
Model 1 unadjusted.
Model 2 adjusted for age, sex, race, baseline diabetes, serum albumin, eGFR (pre-diuretic), systolic blood pressure, and log(NT-proBNP).
RAASi: renin-angiotensinogen-aldosterone system inhibitor.

**Table S3. Associations of Urine and Plasma RAAS Measures with Sodium-Diuretic Efficiency and Fractional Excretion of Sodium-Diuretic Efficiency after Torsemide Administration in 56 Participants with Heart Failure stratified by ACEi/ARB use**

| | Total Sodium Excretion-Diuretic Efficiency | | | | | |
|---|---|---|---|---|---|---|
| | ACEi/ARB non-users | | ACEi/ARB users | | User vs. non-user interaction P-value | |
| RAAS measure (per SD change) | Model 1 ß (95% CI) | Model 2 ß (95% CI) | Model 1 ß (95% CI) | Model 2 ß (95% CI) | Model 1 P-value | Model 2 P-value |
| Urine total renin | **-0.54 (-0.90, -0.18)** | -0.32 (-0.72, 0.083) | -0.43 (-0.88, 0.026) | -0.24 (-0.70, 0.22) | 0.71 | 0.79 |
| Plasma total renin | **-0.45 (-0.82, -0.081)** | **-0.45 (-0.85, -0.043)** | **-0.58 (-1.04, -0.12)** | -0.25 (-0.80, 0.31) | 0.67 | 0.52 |
| Plasma active renin | **-0.67 (-1.16, -0.18)** | -0.53 (-1.09, 0.027) | -0.37 (-0.77, 0.024) | -0.20 (-0.73, 0.32) | 0.35 | 0.31 |
| Urine angiotensinogen | -0.18 (-0.59, 0.22) | 0.025 (-0.42, 0.47) | -0.17 (-0.69, 0.34) | 0.17 (-0.37, 0.70) | 0.98 | 0.67 |
| Plasma angiotensinogen | -0.14 (-0.63, 0.34) | -0.053 (-0.54, 0.44) | -0.28 (-0.74, 0.18) | -0.080 (-0.52, 0.36) | 0.69 | 0.93 |
| Plasma aldosterone | **-0.48 (-0.91, -0.057)** | -0.37 (-0.83, 0.084) | **-0.43 (-0.85, -0.017)** | -0.25 (-0.76, 0.27) | 0.87 | 0.70 |

| | Fractional Excretion of Sodium-Diuretic Efficiency | | | | | |
|---|---|---|---|---|---|---|
| | ACEi/ARB non-users | | ACEi/ARB users | | User vs. non-user interaction P-value | |
| RAAS measure (per SD change) | Model 1 ß (95% CI) | Model 2 ß (95% CI) | Model 1 ß (95% CI) | Model 2 ß (95% CI) | Model 1 P-value | Model 2 P-value |
| Urine total renin | -0.29 (-0.65, 0.074) | -0.33 (-0.76, 0.100) | -0.38 (-0.81, 0.047) | -0.30 (-0.78, 0.18) | 0.75 | 0.94 |
| Plasma total renin | **-0.37 (-0.71, -0.040)** | **-0.49 (-0.88, -0.089)** | **-0.44 (-0.85, -0.026)** | -0.41 (-0.92, 0.099) | 0.81 | 0.82 |
| Plasma active renin | **-0.68 (-1.14, -0.23)** | **-0.78 (-1.31, -0.24)** | -0.22 (-0.57, 0.13) | -0.32 (-0.80, 0.16) | 0.11 | 0.14 |
| Urine angiotensinogen | 0.0067 (-0.37, 0.38) | -0.028 (-0.49, 0.44) | -0.10 (-0.58, 0.38) | -0.13 (-0.70, 0.43) | 0.73 | 0.77 |
| Plasma angiotensinogen | -0.13 (-0.58, 0.31) | -0.22 (-0.74, 0.30) | -0.12 (-0.53, 0.29) | 0.029 (-0.43, 0.49) | 0.95 | 0.47 |
| Plasma aldosterone | **-0.47 (-0.86, -0.078)** | -0.41 (-0.89, 0.072) | -0.038 (-0.46, 0.39) | 0.012 (-0.50, 0.52) | 0.14 | 0.20 |

Urine measures indexed to urine creatinine. SD: standard deviation. CI: confidence interval.
Model 1 unadjusted.
Model 2 adjusted for age, sex, race, baseline diabetes, serum albumin, eGFR (pre-diuretic), systolic blood pressure, and log(NT-proBNP).
ACEi: angiotensin converting enzyme inhibitor; ARB: angiotensin receptor blocker

**Table S4. Associations of Urine and Plasma RAAS Measures with Sodium-Diuretic Efficiency and Fractional Excretion of Sodium-Diuretic Efficiency after Torsemide Administration in 56 Participants with Heart Failure stratified by MRA use**

| | **Total Sodium Excretion-Diuretic Efficiency** | | | | | |
|---|---|---|---|---|---|---|
| | **MRA non-users** | | **MRA users** | | **User vs. non-user interaction P-value** | |
| **RAAS measure (per SD change)** | **Model 1 ß (95% CI)** | **Model 2 ß (95% CI)** | **Model 1 ß (95% CI)** | **Model 2 ß (95% CI)** | **Model 1 P-value** | **Model 2 P-value** |
| Urine total renin | **-0.42 (-0.74, -0.11)** | -0.16 (-0.50, 0.19) | **-0.78 (-1.35, -0.21)** | -0.56 (-1.13, 0.0027) | 0.28 | 0.22 |
| Plasma total renin | -0.39 (-0.78, 0.0029) | -0.34 (-0.76, 0.086) | **-0.60 (-1.08, -0.12)** | -0.39 (-0.93, 0.15) | 0.50 | 0.85 |
| Plasma active renin | **-0.48 (-0.87, -0.091)** | -0.33 (-0.79, 0.13) | **-0.60 (-1.14, -0.057)** | -0.33 (-1.03, 0.38) | 0.73 | 0.99 |
| Urine angiotensinogen | -0.24 (-0.59, 0.11) | 0.015 (-0.35, 0.38) | -0.13 (-0.80, 0.53) | 0.41 (-0.27, 1.08) | 0.78 | 0.27 |
| Plasma angiotensinogen | -0.30 (-0.69, 0.092) | -0.22 (-0.62, 0.17) | 0.25 (-0.46, 0.97) | 0.37 (-0.27, 1.00) | 0.18 | 0.13 |
| Plasma aldosterone | **-0.58 (-0.98, -0.18)** | -0.28 (-0.73, 0.16) | **-0.60 (-1.05, -0.15)** | -0.49 (-1.00, 0.027) | 0.95 | 0.51 |

| | **Fractional Excretion of Sodium-Diuretic Efficiency** | | | | | |
|---|---|---|---|---|---|---|
| | **MRA non-users** | | **MRA users** | | **User vs. non-user interaction P-value** | |
| **RAAS measure (per SD change)** | **Model 1 ß (95% CI)** | **Model 2 ß (95% CI)** | **Model 1 ß (95% CI)** | **Model 2 ß (95% CI)** | **Model 1 P-value** | **Model 2 P-value** |
| Urine total renin | -0.24 (-0.54, 0.063) | -0.23 (-0.60, 0.15) | -0.53 (-1.07, 0.010) | -0.49 (-1.10, 0.12) | 0.35 | 0.47 |
| Plasma total renin | **-0.38 (-0.72, -0.045)** | **-0.43 (-0.83, -0.035)** | -0.38 (-0.82, 0.057) | -0.44 (-0.97, 0.095) | 0.99 | 0.99 |
| Plasma active renin | **-0.36 (-0.73, -0.0018)** | **-0.51 (-0.95, -0.070)** | -0.44 (-0.94, 0.066) | -0.62 (-1.30, 0.052) | 0.82 | 0.74 |
| Urine angiotensinogen | -0.14 (-0.45, 0.16) | -0.15 (-0.53, 0.24) | 0.25 (-0.36, 0.86) | 0.23 (-0.49, 0.95) | 0.26 | 0.33 |
| Plasma angiotensinogen | -0.21 (-0.55, 0.12) | -0.23 (-0.66, 0.20) | 0.28 (-0.33, 0.89) | 0.34 (-0.34, 1.02) | 0.16 | 0.17 |
| Plasma aldosterone | -0.15 (-0.53, 0.23) | -0.12 (-0.57, 0.33) | **-0.46 (-0.90, -0.022)** | -0.53 (-1.07, 0.013) | 0.29 | 0.20 |

Urine measures indexed to urine creatinine. SD: standard deviation. CI: confidence interval.
Model 1 unadjusted.
Model 2 adjusted for age, sex, race, baseline diabetes, serum albumin, eGFR (pre-diuretic), systolic blood pressure, and log(NT-proBNP).
MRA: mineralocorticoid receptor antagonist
